# Lovastatin lactone elicits human lung cancer cell apoptosis *via* a COX-2/PPARγ-dependent pathway

**DOI:** 10.18632/oncotarget.7213

**Published:** 2016-02-05

**Authors:** Udo Walther, Kristin Emmrich, Robert Ramer, Nadine Mittag, Burkhard Hinz

**Affiliations:** ^1^ Institute of Toxicology and Pharmacology, Rostock University Medical Center, Rostock, Germany

**Keywords:** lovastatin lactone, cyclooxygenase-2, peroxisome proliferator-activated receptor γ, apoptosis, human lung cancer cells

## Abstract

Statins (3-hydroxy-3-methylglutaryl coenzyme A [HMG-CoA] reductase inhibitors) are well-established agents to treat hyperlipidemic states. Experimental and epidemiological evidence further implies an anticancer effect of these substances. This study investigates the mechanism underlying human lung cancer cell death by lovastatin and the role of the prostaglandin (PG)-synthesizing enzyme cyclooxygenase-2 (COX-2) in this process. In A549 and H358 lung carcinoma cells the lipophilic prodrug lovastatin lactone led to a concentration-dependent decrease of viability and induction of DNA fragmentation, whereas its HMG-CoA-inhibitory, ring-open acid form was inactive in this respect. Apoptotic cell death by lovastatin was accompanied by high intracellular levels of the lactone form, by upregulation of COX-2 mRNA and protein, as well as by increased formation of peroxisome proliferator-activated receptor γ (PPARγ)-activating PGD_2_ and 15-deoxy-Δ^12,14^-PGJ_2_. Cells were significantly less sensitive to lovastatin-induced apoptotic cell death, when the expression or activity of COX-2 was suppressed by siRNA or by the COX-2 inhibitor NS-398. Apoptosis by lovastatin was likewise reversed by the PPARγ antagonist GW9662. Fluorescence microscopy analyses revealed a lovastatin-induced cytosol-to-nucleus translocation of PPARγ that was inhibited by NS-398. Collectively, this study demonstrates COX-2 induction and subsequent COX-2-dependent activation of PPARγ as a hitherto unknown mechanism by which lovastatin lactone induces human lung cancer cell death.

## INTRODUCTION

Belonging to the most commonly prescribed drugs worldwide, statins are therapeutically used to treat primary and secondary hypercholesterolemia. Statins inhibit 3-hydroxy-3-methylglutaryl coenzyme A (HMG-CoA) reductase, an early and rate-limiting enzyme of cholesterol synthesis, thereby preventing the conversion of HMG-CoA to mevalonate, and reducing the levels of mevalonate and its downstream products. Statins are administered in its active ring-open hydroxy-acid form (e.g., pravastatin, atorvastatin) or as inactive lactone prodrugs (lovastatin, simvastatin) with the latter group of drugs becoming metabolized to a ring-open hydroxy-acid form that inhibits HMG-CoA reductase activity (for review see [1]).

Besides their use as cholesterol-lowering agents, statins are currently considered and evaluated as potential drugs for cancer therapy [2]. Accordingly, several epidemiological studies have proven an association between statin use and diminished cancer incidence [3-5] as well as mortality [6]. In case of lung cancer, a retrospective case-control study has found an association of statin use for > 6 months with a 55% risk reduction [7]. In addition, *in vitro* experiments with cancer cells revealed statins to exhibit pronounced antiproliferative [8, 9], proapoptotic [10, 11], anti-invasive [12-14] and anti-angiogenic effects [15-17].

However, conflicting data have been published concerning the contribution of lactone and acid forms to the anticancerogenic statin action. On the one hand, several studies have associated such effects with decreased formation of the mevalonate downstream products farnesyl pyrophosphate and geranylgeranyl pyrophosphate by ring-open acid forms of statins. In fact, both products are essential regulators of membrane localisation and function of small G proteins [18] that confer mitogenic [19], adhesive and invasive properties [20] of cancer cells. On the other hand, the dogma of the ring-open form being the sole active configuration of statins has been challenged. Accordingly, lovastatin lactone was shown to elicit growth inhibitory effects on human breast cancer cells by inhibition of the proteasome, whereas pravastatin, a ring-open and therefore direct HMG-CoA reductase-inhibitory statin with a structure and potency similar to lovastatin acid, was inactive in both respects [21]. This and sequential studies [22, 23] have raised the question whether physicochemical properties (i.e., lipophilicity that determines the ability to pass cellular membranes) might explain the differential impact of statins on cancer growth. However, despite the fact that lovastatin lactone elicits proteasome inhibition [21-23], the exact mechanism underlying its cytotoxic and proapoptotic action on cancer cells is far from being understood.

In past years upregulation of the prostaglandin (PG)-synthesizing enzyme cyclooxygenase-2 (COX-2) has emerged as a proapoptotic mechanism shared by various antitumorigenic compounds including chemotherapeutics [24-27], cannabinoids [28-31], endocannabinoid-like substances [32] as well as the analgesic celecoxib [33]. In this context, several studies indicated COX-2-derived PGD_2_ and 15-deoxy-Δ^12,14^-PGJ_2_ (15d-PGJ_2_) to evoke COX-2-dependent apoptosis by activating the transcription factor peroxisome proliferator-activated receptor γ (PPARγ) [26, 29, 31, 33-36]. Notably, statins likewise induce the expression of COX-2 [37-39] or activate PPARγ [40] in a variety of cell types. However, a causal link of these targets to statin-induced cancer cell apoptosis has not been established so far.

The present study therefore investigates a potential contribution and coordinated action of COX-2 and PPARγ within the lovastatin lactone-induced apoptosis of human lung cancer cells. Here we present evidence for a hitherto unknown statin-induced proapoptotic pathway involving initial upregulation of COX-2 and a subsequent activation of PPARγ by de novo synthesized COX-2-dependent PGs.

## RESULTS

### Impact of lovastatin lactone and lovastatin acid on apoptotic lung cancer cell death

**Figure 1:**
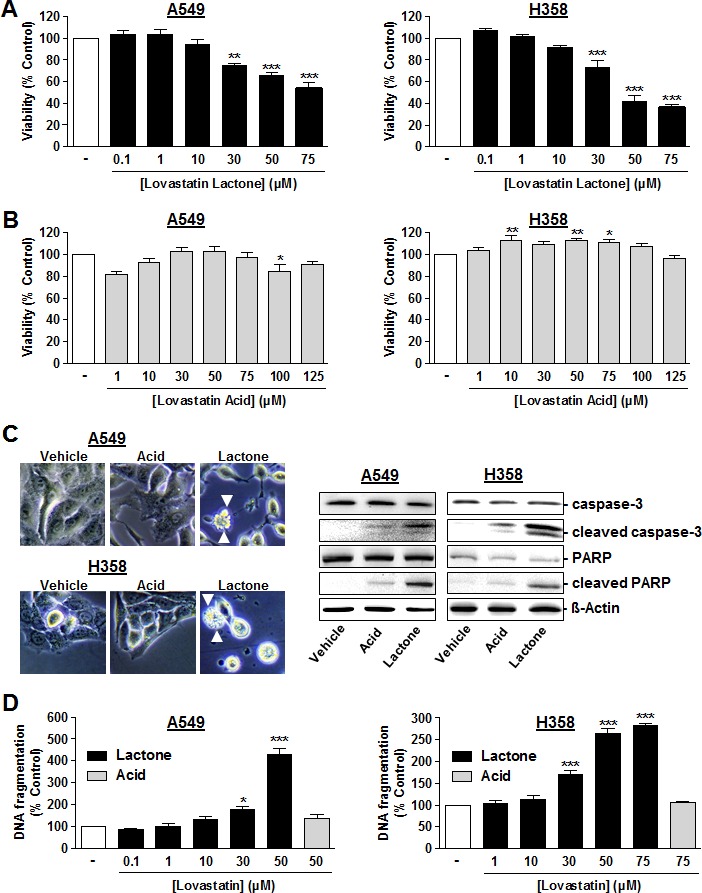
Effect of lovastatin lactone and lovastatin acid on cellular viability and apoptosis of A549 and H358 cells **A.**, **B.** A549 cells (left panels) or H358 cells (right panels) were incubated with the indicated concentrations of lovastatin lactone **A.** or lovastatin acid **B.** for 48 h (WST-1 test). **C.**, *left* Microscopic images were taken following a 24-h incubation period of A549 cells or H358 cells with vehicle, 50 μM (A549), or 75 μM (H358) lovastatin lactone (lactone) or lovastatin acid (acid), respectively. Arrowheads indicate cells with characteristic apoptotic morphology. **C.**, *right* Western blots analyses of caspase-3, cleaved caspase-3, PARP and cleaved PARP in response to lovastatin lactone and acid (both at 50 μM in A549 and at 75 μM in H358) following a 48-h incubation period. Cleaved caspase-3 appears as a 17 kDa band (lower band). Images of Western blot analyses depict one representative Western blot result of 4-8 independent experiments. **D.** Quantification of DNA fragmentation following a 48-h incubation of A549 cells (left panel) or H358 cells (right panel) with the indicated concentrations of lovastatin lactone (black bars) or lovastatin acid (gray bars). Percent control represents comparison with vehicle-treated cells (100%) in the absence of test substance. Values are mean ± SEM of *n* = 3 - 10 (A, left), *n* = 8-13 (A, right), *n* = 5 - 11 (B, left), *n* = 5 - 15 (B, right), *n* = 4 (D, left), *n* = 3-4 (D, right). *P < 0.05; **P < 0.01; ***P < 0.001 *vs*. corresponding vehicle control; one-way ANOVA plus Dunnett test (A; B; D). In histograms the vehicle control bars do not contain SEM with respect to different numbers of experiments that were carried out with different concentrations. However, statistical evaluation was only carried out using vehicle controls of the actual experiment.

Analysis of the effects of lovastatin on the viability of A549 and H358 cells revealed lovastatin lactone (Figure 1A) but not the corresponding acid form (Figure 1B) to exhibit concentration-dependent cytotoxic properties. IC_50_ values of lovastatin lactone's effect on viability were 76.7 μM (A549) and 45.2 μM (H358), respectively. Lovastatine lactone at 50 μM (A549) and 75 μM (H358) elicited characteristic apoptotic features such as membrane blebbing that were not observed in A549 and H358 cells treated with equimolar concentrations of lovastatin acid (Figure 1C, left side). In agreement with these observations, additional apoptotic parameters such as caspase-3 were triggered by lavostatin lactone, whereas the acid form only faintly induced caspase-3 activation in both cell lines (Figure 1C, right side, upper 2 blots). To confirm the caspase-3-dependent apoptotic pathway, we next analyzed cleavage of the DNA repair protein and caspase-3 substrate, poly(ADP-ribose) polymerase (PARP). In line with the cleavage pattern of caspase-3, the lactone form induced PARP cleavage to a much larger extent than the acid form (Figure 1C, right side, blots in line 3 and 4).

Quantification of DNA fragmentation as a further apoptotic characteristic revealed a concentration-dependent DNA fragmentation by lovastatin lactone that was not detectable in cells treated with lovastatin acid (Figure 1D).

### Extra- and intracellular concentrations of lovastatin lactone and acid following incubation of cells with either form

To determine the extent of extracellular and intracellular hydrolysis of lovastatin lactone as well as its uptake as unhydrolyzed lipophilic form, time-course experiments with lovastatin lactone-treated A549 and H358 cells were performed. Using the same experimental setting, comparative experiments were carried out with cells incubated with an equimolar concentration of lovastatin acid.

**Figure 2:**
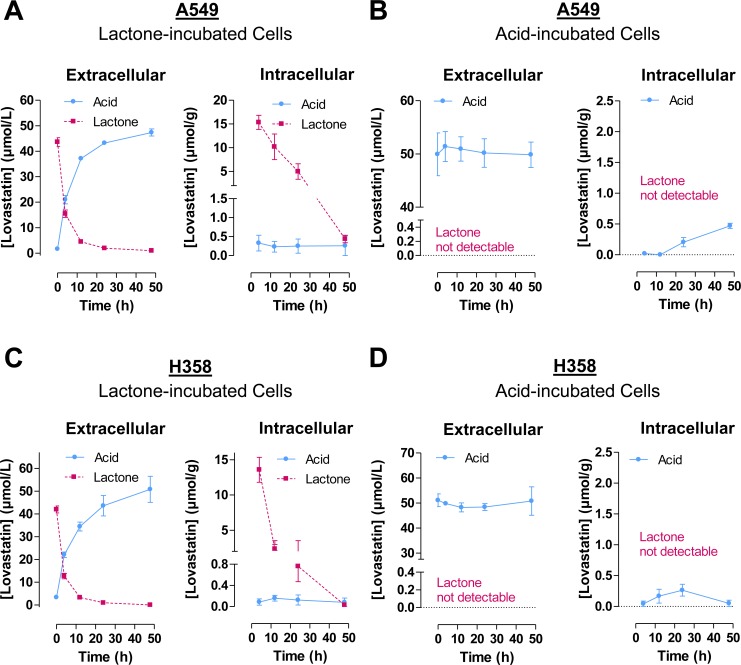
Time-course of extracellular and intracellular concentrations of lovastatin lactone and acid following addition of either lovastatin lactone or lovastatin acid to A549 or H358 cells Cells were incubated with 50 μM lovastatin lactone **A.**, **C.** or acid **B.**, **D.** for up to 48 h. Concentrations of lovastatin lactone and acid were measured after the indicated time intervals in the medium and the cellular fraction by HPLC as described under Materials and Methods. For intracellular levels, lovastatin lactone and acid levels determined by HPLC were normalized to the respective total cellular protein amounts. Lovastatin lactone and acid measured in cell culture supernatants are presented as un-normalized raw concentrations. Values are mean ± SEM of *n* = 3 (A; B, left panel; D), *n* = 2 - 3 (B, right panel), *n* = 3 - 4 (C).

As shown in Figure 2A, 2C, left, extracellular lovastatin lactone measured in cell culture supernatants became hydrolyzed to its open-ring acid form in a time-dependent manner. However, profound concentrations of lovastatin lactone were measured in cell lysates after 4 h, proving a substantial uptake of the lipophilic form (Figure 2A, 2C, right). Intracellular lovastatin lactone concentrations decreased over time reaching 2.9% and 0.2% of the 4-h value in A549 and H358 cells after a 48-h incubation period (Figure 2A, 2C, right). By contrast, HPLC analysis yielded low intracellular concentrations of lovastatin acid, which did not rise concomitantly with the time-dependent intracellular decrease of the lactone form.

Incubation of cells with the acid form of lovastatin resulted in constant extracellular concentrations of this compound and no measurable lactone levels in cell culture supernatants of both A549 and H358 cells (Figure 2B, 2D, left).

Remarkably, intracellular concentrations of the acid (Figure 2B, 2D, right) were approximately in the same range as the acid concentrations measured in lactone-treated cells (Figure 2A, 2C, right). Thus, lovastatin acid was ranging between 0 and 0.5 μmol/g protein in A549 cells and between 0 and 0.3 μmol/g protein in H358 cells following treatment with the acid form (Figure 2B, 2D, right). In lactone-treated cells the acid form ranged between 0.2 and 0.3 μmol/g protein (A549) and between 0.1 and 0.2 μmol/g protein (H358), respectively (Figure 2A, 2C, right).

### Impact of lovastatin lactone on COX-2 and PPARγ expression

In view of several studies indicating COX-2-derived PGs to confer COX-2-dependent apoptosis by activating the transcription factor PPARγ, the impact of lovastatin lactone on COX-2 was assessed in A549 and H358 cells next.

**Figure 3:**
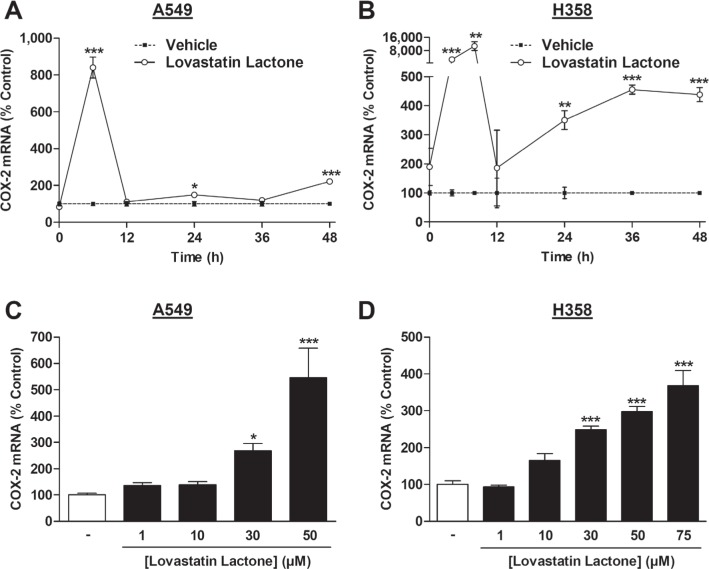
Effect of lovastatin lactone on COX-2 mRNA expression in A549 and H358 cells **A.**, **B.** Real-time RT-PCR analyses of the effect of 50 μM (A, A549) or 75 μM (B, H358) lovastatin lactone on COX-2 mRNA expression over a 48-h incubation period. **C.**, **D.** Concentration-dependent effect of lovastatin lactone on COX-2 mRNA expression after a 48-h incubation period. Percent control represents comparison with vehicle-treated cells (100%, dashed line in A, B) in the absence of test substances. Values are mean ± SEM of *n* = 3 - 4 (A; B), *n* = 6 - 7 (C) and *n* = 4 (D). *P < 0.05; **P < 0.01; ***P < 0.001 *vs*. corresponding vehicle control; Student's *t* test (A; B) or one-way ANOVA plus Dunnett test (C; D).

In fact, lovastatin lactone caused an upregulation of COX-2 mRNA levels in a time-dependent manner (Figure 3A, 3B) with a rapid induction of COX-2 mRNA after a 6-h (A549) or 4- and 8-h incubation (H358) and significant increases at 48 h. To measure mRNA regulations at the time of functional implications, i.e., loss of viability and induction of apoptosis, the concentration-dependency of this effect was evaluated following a 48-h incubation with the substance, yielding a concentration-dependent induction of COX-2 mRNA by lovastatin lactone (Figure 3C, 3D).

**Figure 4:**
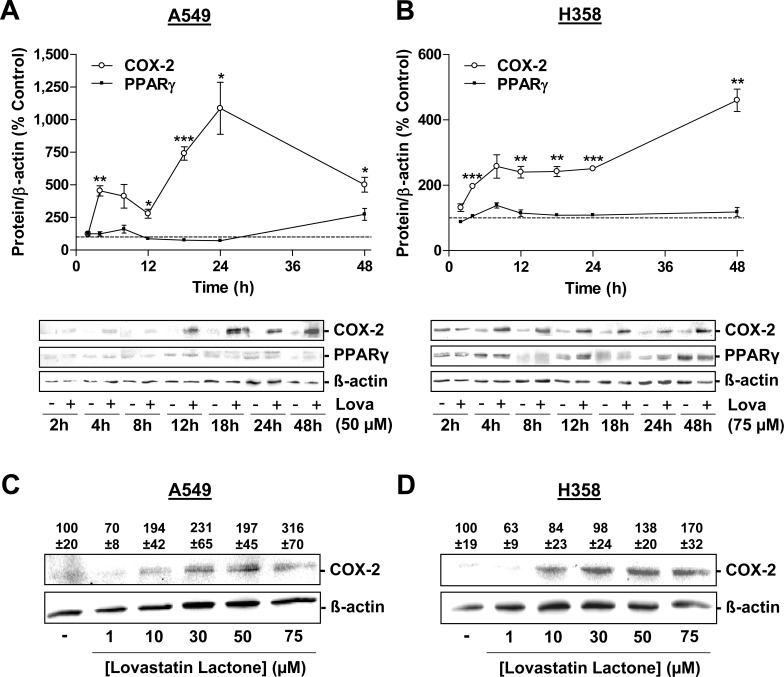
Effect of lovastatin lactone on COX-2 and PPARγ protein expression in A549 and H358 cells **A.**, **B.** Western blot analysis of the effect of 50 μM (A, A549) and 75 μM (B, H358) lovastatin lactone on COX-2 and PPARγ protein expression over a 48-h incubation period. **C.**, **D.** Concentration-dependent effect of lovastatin lactone on COX-2 protein expression following a 24-h incubation period of A549 (C) and H358 (D) cells. Densitometric evaluations of Western blots are presented as percent of vehicle control (100%) in the charts (A,B; vehicle indicated as dashed line) or above the blots (C; D). All densitometric values were normalized to Δ-actin. Values are mean ± SEM of *n* = 3- 4 (A), *n* = 4 - 8 (B) or *n* = 4 (C; D) blots. *P < 0.05; **P < 0.01; ***P < 0.001 *vs*. corresponding vehicle control; Student's *t* test (A; B).

In line with this data, incubation of cells with lovastatin lactone was associated with substantial increases in COX-2 protein levels. In experiments addressing the time-dependency of this action over a 48-h incubation period, lovastatin lactone elicited increases of COX-2 protein within 4 h in both cell lines (Figure 4A, 4B). A concentration-dependency of COX-2 upregulation by lovastatin was observed in both A549 (Figure 4C) and H358 cells (Figure 4D).

Finally, analysis of a potential time-dependent alteration of PPARγ, the primary target of COX-2-dependent PGs in evoking apoptosis, revealed no significant alteration on the level of protein expression (Figure 4A, 4B).

### Impact of mevalonic acid on lovastatin lactone-induced apoptotic cell death and COX-2 expression

**Figure 5:**
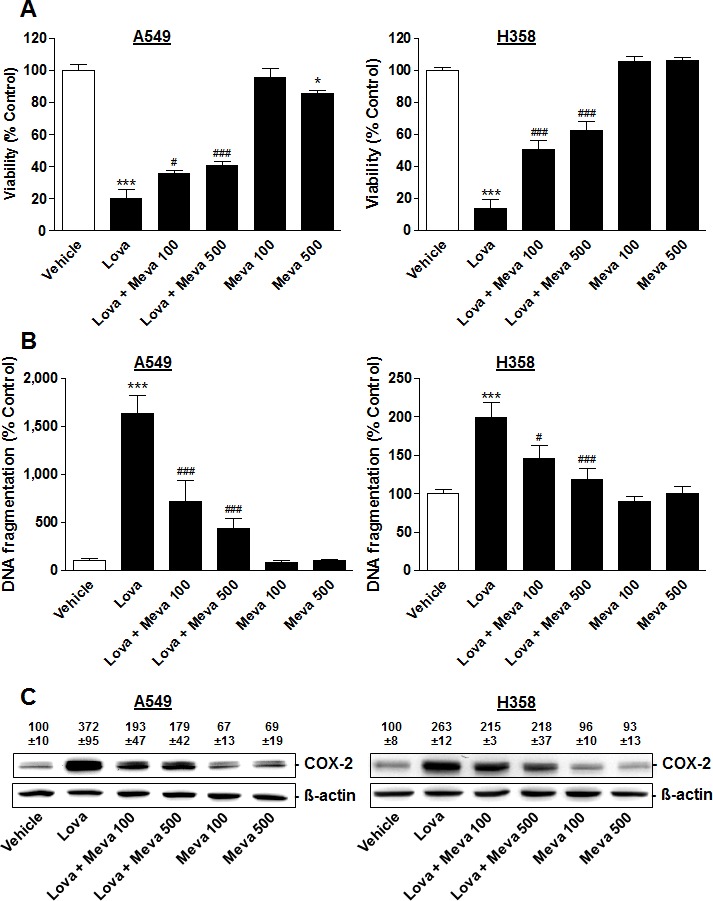
Impact of mevalonic acid on modulation of viability, DNA fragmentation and COX-2 expression by lovastatin lactone in A549 and H358 cells Mevalonic acid at 100 or 500 μM was added 1 h prior to addition of lovastatin lactone at 50 μM (A549) or 75 μM (H358) and incubation was continued for another 48 h (A549) or 24 h (H358). **A.**, **B.** Viability (WST-1 test) and DNA fragmentation analyses. **C.** Western blot analyses of COX-2 expression. Densitometric evaluations of Western blots are presented as percent of vehicle control (100%). All densitometric values were normalized to Δ-actin. Values are mean ± SEM of *n* = 12 (A), *n* = 4 (B, left; C, left), *n* = 8 (B, right) or *n* = 3 (C, right). *P < 0.05; ***P < 0.001 *vs*. corresponding vehicle control; ^#^*P* < 0.05; ^###^*P* < 0.001 *vs*. lovastatin lactone; one-way ANOVA plus Bonferroni test.

To determine whether the lovastatin lactone-elicited increases of DNA fragmentation and COX-2 expression were due to inhibition of HMG-CoA reductase, both effects were investigated in the presence of mevalonic acid, the direct product of HMG-CoA reductase. Mevalonic acid tested at 100 μM has been reported to sufficiently block statin effects [11, 15, 17]. However, mevalonic acid only partially prevented cytotoxicity (Figure 5A), DNA fragmentation (Figure 5B) and COX-2 expression (Figure 5C) by lovastatin lactone in both A549 and H358 cells, even when used at a concentration of 500 μM. Mevalonic acid did not inhibit DNA fragmentation itself (Figure 5B). A minor decrease of viability was even observed in the presence of the 500-μM concentration in A549 cells, which was, however, not found in H358 cells (Figure 5A).

### Impact of lovastatin lactone on PG production

**Figure 6:**
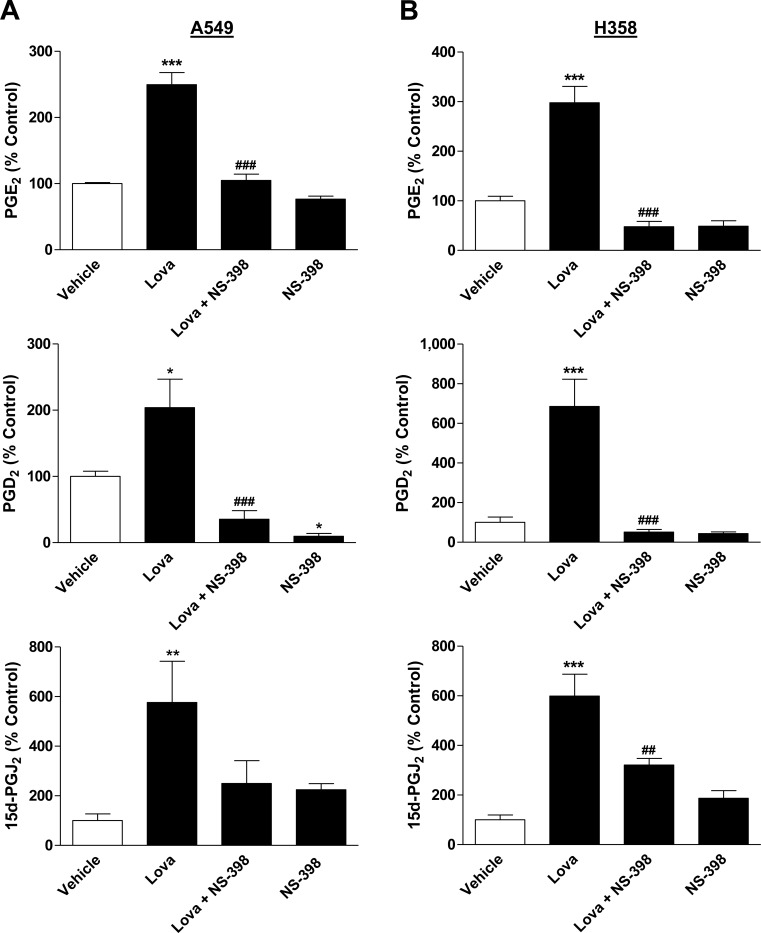
Effect of lovastatin lactone on PG synthesis by A549 and H358 cells **A.**, **B.** Cells were treated with vehicle or lovastatin lactone at 50 μM (A, A549) or 75 μM (B, H358) for 24 h in the presence or absence of NS-398 (1 μM) that was added to the cells 1 h prior to the incubation with lovastatin lactone. PG levels were determined in cell culture media and were normalized to total cellular protein amounts. Percent control represents comparison with vehicle-treated cells (100%) in the absence of test substances. Basal, protein-unnormalized PG levels in cell culture media of vehicle-treated cells were as follows: PGE_2_, 98.27 ± 5.97 pM (A549); PGE_2_, 39.05 ± 1.33 pM (H358); PGD_2_, 34.84 ± 9.13 pM (A549); PGD_2_, 16.60 ± 3.19 pM (H358); 15d-PGJ_2_, 14.76 ± 4.75 pM (A549); 15d-PGJ_2_, 18.65 ± 2.80 pM (H358). Values are mean ± SEM of *n* = 4 (A, PGE_2_; B, 15d-PGJ_2_), *n* = 8 (A, 15d-PGJ_2_), *n* = 7 - 8 (A, PGD_2_), *n* = 3 - 4 (B, PGD_2_) and *n* = 2 - 4 (B, PGE_2_ [2 values of the group treated with NS-398 were below the limit of PGE_2_ detection]). *P < 0.05; **P < 0.01; ***P < 0.001 *vs*. corresponding vehicle control; ^##^*P* < 0.01; ^###^*P* < 0.001 *vs*. lovastatin lactone, one-way ANOVA plus Bonferroni test.

Additional experiments were performed to investigate the production of PGs through treatment of cells with lovastatin lactone. To evaluate whether a potential upregulation of PG production was causally linked to increased COX-2 expression, these experiments likewise included combined incubation of cells with lovastatin lactone and the selective COX-2 inhibitor NS-398. As shown in Figure 6A, 6B, lovastatin lactone induced significant releases of PGE_2_, PGD_2_ and 15d-PGJ_2_ in both cell lines with all increases being sensitive to NS-398.

### Impact of COX-2 and PPARγ on lovastatin lactone-induced apoptotic cell death

**Figure 7:**
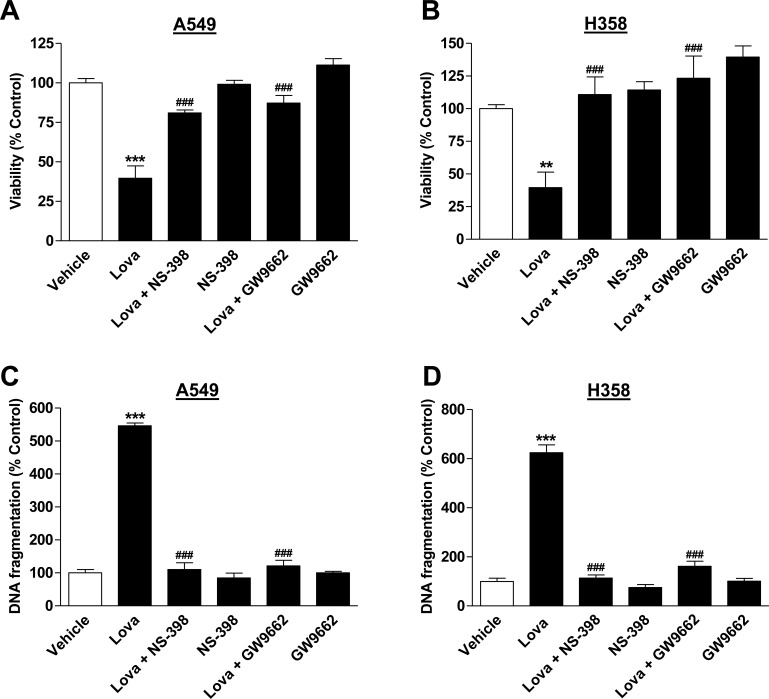
Impact of NS-398 and GW9662 on lovastatin lactone-induced apoptotic cell death Viability (**A.**, **B.**; WST-1 test) and DNA fragmentation (**C.**, **D.**; DNA fragmentation assay) of A549 and H358 cells. NS-398 (1 μM) or GW9662 (10 μM) were added to the cells 1 h prior to lovastatin lactone (50 μM in A549; 75 μM in H358) or vehicle and incubation was continued for another 48 h (WST-1 test) or 24 h (DNA fragmentation). Percent control represents comparison with vehicle-treated cells (100%) in the absence of test substances. Values are mean ± SEM of *n* = 13 - 14 (A), *n* = 9 - 10 (B), *n* = 4 (C; D), **P < 0.01; ***P < 0.001 *vs*. vehicle control; ^###^*P* < 0.001 *vs*. lovastatin lactone, one-way ANOVA plus Bonferroni test.

To investigate a potential involvement of COX-2 and PPARγ in lovastatin lactone-induced apoptotic cell death, experiments using NS-398 and the PPARγ antagonist GW9662 were performed. As shown in Figure 7, NS-398 and GW9662 inhibited both toxicity (Figure 7A, 7B) and DNA fragmentation (Figure 7C, 7D) by lovastatin lactone in each cell line.

**Figure 8:**
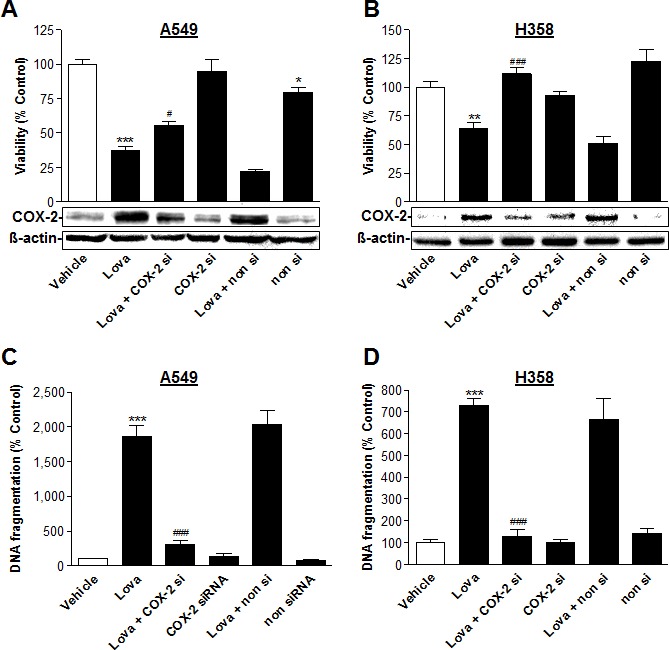
Impact of COX-2 siRNA on lovastatin lactone-induced apoptotic cell death of A549 and H358 cells Effect of COX-2 siRNA on cellular viability (**A.**, **B.**, upper panel; WST-1 test), COX-2 protein expression (**A.**, **B.**, lower panel; Western blot analyses) and DNA fragmentation (C., D.; DNA fragmentation assay) in the presence or absence of 50 μM (A,C; A549) or 75 μM (B,D; H358) lovastatin lactone. Cells were incubated with lovastatin lactone or vehicle for 48 h (A; B) or 24 h (C; D) Transfection with COX-2 siRNA (2.5 μg/ml) or the respective equal concentration of non-silencing siRNA was performed 24 h prior to addition of test compounds to the cells. β-actin was used as loading control for Western blot analysis. Percent control represents comparison with vehicle-treated cells (100%) in the absence of test substances. Values are mean ± SEM of *n* = 4 (A), *n* = 6 (B), *n* = 3 - 4 (C; D). *P < 0.05; **P < 0.01; ***P < 0.001 *vs*. vehicle control; ^#^*P* < 0.05; ^###^*P* < 0.001 *vs*. lovastatin lactone; one-way ANOVA plus Bonferroni test.

To further substantiate the role of de novo expressed COX-2 in lovastatin lactone-induced apoptotic cell death, transfection experiments were performed using siRNA targeting COX-2. Transfection of cells with COX-2 siRNA was shown to interfere with lovastatin lactone-induced COX-2 protein levels (Figure 8A, 8B, Western blot images, lower panel) and significantly inhibited toxicity (Figure 8A, 8B, histograms, upper panel) and DNA fragmentation (Figure 8C, 8D) elicited by lovastatin lactone in both cell lines.

### Role of COX-2 in PPARγ activation by lovastatin lactone

On the basis of the data showing a lovastatin lactone-induced upregulation of COX-2 and a functional role of COX-2 and PPARγ in its proapoptotic action, a potential coordinated action of COX-2 and PPARγ was investigated next. To this end, experiments were performed to clarify whether a combination of lovastatin lactone and the COX-2 inhibitor NS-398 may abrogate the lactone-induced PPARγ activation.

**Figure 9:**
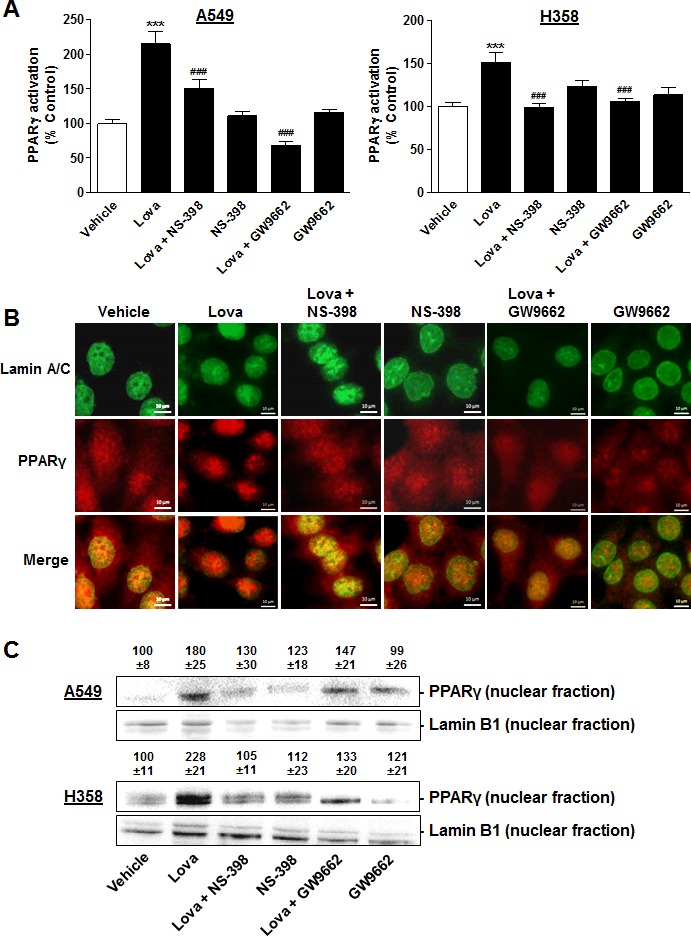
Impact of COX-2 and PPARγ inhibition on PPARγ translocation in A549 and H358 cells **A.**, **B.** Fluorescence microscopic analyses in cells treated with lovastatin lactone at 50 μM (A549, A, left panel) or 75 μM (H358, A, right panel, images in B) in the presence or absence of NS-398 (1 μM) and GW9662 (10 μM). Cells were pretreated with NS-398 or GW9662 1 h prior to addition of lovastatin lactone. Thereafter, incubation was continued for another 12 h (A549) or 24 h (H358). PPARγ activation was quantified by measuring colocalization of PPARγ and nuclear regions. Nuclear regions were identified *via* visualization of lamin A/C by an antibody that was stained by a goat anti-mouse Alexa Fluor^®^ 488 labelled secondary IgG (green dye). PPARγ was stained by antibody binding and subsequent goat anti-rabbit Alexa Fluor^®^ 555 labelled secondary IgG (red dye). Pictures show representative immunocytochemical images of PPARγ and nuclei (lamin A/C) in H358 cells (B). Percent control (A) represents mean ± SEM of *n* = 20 nuclei per sample for each cell line. ***P < 0.001 *vs*. vehicle control; ^###^*P* < 0.001 *vs*. lovastatin lactone; one-way ANOVA plus Bonferroni test. **C.** Western blot analysis of PPARγ protein levels in nuclear fractions of cells treated with vehicle or lovastatin lactone at 50 μM for 18 h (A549) or 75 μM for 24 h (H358). Values above the blots indicate densitometric analysis given as percent control ± SEM in comparison with vehicle-treated cells (100%) in the absence of test substances normalized to the nuclear protein lamin B1 of *n* = 4 (A549) or *n* = 5 (H358) experiments.

In a first approach, cytosol-to-nucleus translocation of PPARγ, a reliable marker of PPARγ activation [41-43], was assessed using fluorescence microscopy. According to Figure 9A, 9B, a profound translocation of PPARγ to nuclear regions became evident when cells were treated with lovastatin lactone. In both cell lines tested the nuclear accumulation of PPARγ by lovastatin lactone was significantly suppressed by the COX-2 inhibitor NS-398. Furthermore, a complete reversal of lovastatin lactone-induced cytosol-to-nucleus translocation of PPARγ was observed when cells were coincubated with the PPARγ antagonist GW9662 indicating PPARγ ligand crosslinking to be involved in this response.

In a second approach, nuclear PPARγ protein levels from A549 and H358 cells were investigated by Western blot analyses of proteins in nuclear fractions. Again, lovastatin lactone was found to increase PPARγ protein levels in nuclear fractions with the respective upregulation being sensitive to both NS-398 and GW9662 (Figure 9C).

## DISCUSSION

**Figure 10:**
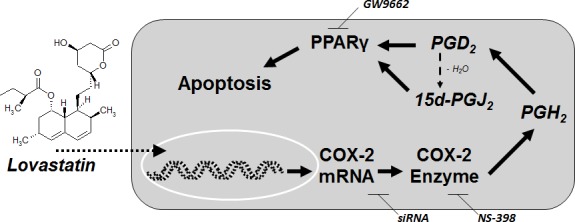
Proposed mechanism underlying the proapoptotic action of lovastatin lactone on lung cancer cells Incubation of lung cancer cells with lovastatin lactone results in profound intracellular levels of the unchanged lactone form. Lovastatin lactone induces a profound upregulation of COX-2 mRNA and protein expression resulting in increases of PGD_2_ and 15d-PGJ_2_, two well-established activators of the transcription factor peroxisome proliferator-activated receptor γ (PPARγ) that elicits apoptosis.

The present study demonstrates induction of COX-2 expression and subsequent activation of PPARγ by COX-2-derived PGs as key events within the proapoptotic action of lovastatin lactone on human lung cancer cells (for summary see Figure 10).

There are several lines of evidence supporting this pathway. First, lovastatin lactone caused a profound upregulation of COX-2 mRNA and protein expression in the lung cancer cell lines A549 and H358. Second, treatment of both cell lines with lovastatin lactone resulted in increases of PGE_2_, PGD_2_ and 15d-PGJ_2_ that were sensitive to NS-398, thus indicating a functionally active COX-2 enzyme. Third, specific inhibition of COX-2 and PPARγ with small molecules suppressed lovastatin lactone-induced apoptotic cell death. The same pattern was observed, when COX-2 was suppressed posttranscriptionally using an siRNA approach. Fourth, lovastatin lactone-induced translocation of PPARγ from cytosol to nucleus, an established marker of PPARγ activation [41-43], was inhibited by NS-398 suggesting COX-2-dependent PGs generated through lovastatin lactone treatment to induce the observed activation of PPARγ. In line with this notion, another study from our group has recently shown that exogenously added PGD_2_ and 15d-PGJ_2_ elicit PPARγ translocation and PPARγ-dependent apoptosis in A549 and H358 cells, whereas PGE_2_ left both events virtually unaltered [33]. These data are in good agreement with other studies demonstrating anticancerogenic effects of PGD_2_ and 15d-PGJ_2_ to occur *via* PPARγ [26, 29, 34-36].

Clearly, the concentrations of lovastatin lactone causing COX-2 induction and DNA fragmentation exceed plasma concentrations of lovastatine lactone, which have been reported to reach a maximum of 0.02 μM after single-dose administration of 80 mg lovastatin to human volunteers [44]. However, in a dose-escalating trial of lovastatin in patients with advanced malignancies, lovastatin administered orally every 6 h for 96 h in 4-week cycles in doses ranging from 10 mg/m^2^ to 412 mg/m^2^ caused peak plasma bioactivity levels of 0.06 to 12.3 μM [45]. In the same investigation, a dose-limiting toxicity was not reached and there were no clinically significant increases in creatine phosphokinase or serum hepatic aminotransferases levels. Noteworthy, high intracellular concentrations may be achieved *in vivo* through longer exposure times. Accordingly, cancer patients receive repeated treatment over weeks or months resulting in cumulative effects of the respective chemotherapy or radiation therapy [46, 47].

Concerning the upstream events conferring increased COX-2 expression by lovastatin lactone, it is tempting to speculate that the previously reported lovastatin lactone-induced inhibition of the proteasome [21-23], which is triggered by its electrophilic carbonyl function, may play a role in this context. As a matter of fact, inhibition of the proteasome has been associated with upregulation of COX-2 expression [48, 49]. In case of the proteasome inhibitors MG132, PSI-1 and lactacystin, induction of COX-2 expression was shown to occur *via* enhanced gene transcription rather than prevention of protein degradation in diverse cells including A549 [49].

Our data indicating the lactone but not the acid form of lovastatin to elicit apoptotic death of lung cancer cells are in line with the work of Rao et al. [21] that challenged the dogma of the ring-open form being the sole active form of lovastatin. In this investigation lovastatin lactone was shown to induce growth inhibitory effects on human breast cancer cells by inhibition of the proteasome, whereas pravastatin, a ring-open and therefore direct HMG-CoA reductase-inhibitory statin with a structure and potency similar to lovastatin acid, did not elicit comparable effects [21]. In another study, mevastatin, which is likewise a statin prodrug with closed-ring structure, induced degenerative changes and reduced viability of differentiated murine neuroblastoma cells by inhibiting proteasome activity, whereas pravastatin neither affected degeneration and viability nor proteasome activity [50]. However, apart from these studies substantiating our observations, the cytotoxic action of the lactone form toward cancer cells appears to be a cell type-dependent phenomenon. Thus, in contrast to the data presented here, lovastatin acid has been previously shown to elicit apoptosis in various pediatric cancer cells and squamous cell carcinomas, whereas the lactone form was inactive in this respect [51].

A reason for the differential effects of lactone and acid forms on viability may lie in their diverse and variable uptake mechanisms by different cell types. In this context it is worthy to note that lovastatin lactone is almost three orders of magnitude more lipophilic than its active ring-open hydroxy-acid form [1, 52]. As a matter of fact, increased lipophilicity of the lactone is reflected by its higher potential to cross cellular membranes non-selectively by passive diffusion as compared to its ring-open hydroxy-acid form [1]. As shown for hepatocytes, lipophilic statins enter the cells by passive diffusion, whereas hydrophilic statins require a carrier-mediated uptake [1, 53].

In the present investigation HPLC analyses of lovastatin lactone-treated cells revealed profound intracellular levels of the lactone with initial concentrations being 51- (A549) or 136-fold (H358) above the corresponding intracellular concentrations of the ring-open acid form, the hydrolysis product of the lactone prodrug. These data are in line with a study by Kumar et al. [50] that even exclusively found the ring-closed form of mevastatin in neuronal cells incubated with the lipophilic prodrug. On the other hand, lovastatin lactone was not detected in both A549 and H358 cells treated with the acid form. On the basis of these data proving a substantial uptake of the lipophilic prodrug form, it is most likely that the lactone itself elicits COX-2 expression and apoptotic response of lactone-treated cells. This view is substantiated by the finding that virtually identical intracellular levels of the acid form were measured in cells treated with equimolar concentrations of either lactone or acid. Thus, if these low intracellular lovastatin acid levels, probably resulting from intracellular conversion, were primarily responsible for apoptosis induction by the lactone, incubation of cells with lovastatin acid should be likewise expected to elicit apoptosis, which could not be confirmed here.

In apparent contrast to these considerations, mevalonic acid, the product of HMG-CoA-reductase-catalyzed reaction, was shown to suppress both apoptotic response and COX-2 expression by the lactone. On the one hand, these data imply at least to some extent a role of HMG-CoA reductase inhibition in both actions of the lactone. Thus, intracellular generation of active inhibitors of HMG-CoA reductase, other than the ring-open hydroxy-acid form, may contribute to the effects of lovastatin lactone observed in this study. These metabolites may derive from sequential oxidation and hydrolysis of the respective lactone rather than from oxidation of the active ring-open hydroxy-acid form (for review see [1]) and have been previously found to circulate in serum of lactone prodrug-treated subjects [54-56]. On the other hand, mevalonic acid may also interfere with events prior to lovastatin lactone-induced COX-2 expression and apoptosis. Accordingly, Rao et al. [21] have shown that mevalonate abrogates the lovastatin lactone-induced inhibition of the proteasome and G_1_ arrest. In line with this notion, mevalonate completely abrogated apoptosis by lactacystin, an established proteasome inhibitor [21]. A few years later, Kumar et al. [50] using neuroblastoma cells were able to demonstrate that mevalonic acid lactone completely prevents mevastatin-induced degeneration and decreased viability by reducing the uptake of mevastatin and by blocking its action on proteasome activity.

Collectively, this study demonstrates a hitherto unknown proapoptotic mechanism of lovastatin lactone comprising upregulation of COX-2 expression and activation of PPARγ by de novo synthesized COX-2-derived PGs. Moreover, our results challenge the wide-spread view of the acid form being the sole active form of statins.

## MATERIALS AND METHODS

### Materials

NS-398 was purchased from Alexis Deutschland GmbH (Grünberg, Germany). Aprotinin, Δ-glycerophosphate, ethylenediaminetetraacetic acid (EDTA), leupeptin, lovastatin lactone, luminal, mevastatin, p-coumaric acid, phenylmethylsulfonyl fluoride (PMSF), (R)-mevalonic acid lithium salt, sodium molybdate and sodium orthovanadate were obtained from Sigma-Aldrich (Taufkirchen, Germany). 4-(2-hydroxyethyl)-1-piperazineethanesulfonic acid (HEPES) was from Ferak (Berlin, Germany). Dimethyl sulfoxide (DMSO), dithiothreitol (DTT), glycerol, p-nitrophenylphosphate, sodium chloride, sodium dodecylsulfate (SDS) and sodium fluoride were from AppliChem (Darmstadt, Germany) and GW9662 and Nonidet^®^ P-40 from Enzo Life Sciences (Lörrach, Germany). Lovastatin hydroxy acid, sodium salt, was provided from Toronto Research Chemical (Toronto, Canada) and Triton^®^ X-100, acetonitrile (LC-MS grade) and trifluoroacetic acid (analytical grade) from Roth (Karlsruhe, Germany). Penicillin-streptomycin was from Invitrogen (Darmstadt, Germany). Dulbecco's Modified Eagle's medium (DMEM) with 4 mM L-glutamine and 4.5 g/L glucose was from Lonza (Cologne, Germany). Phosphate-buffered saline (PBS) and fetal calf serum (FCS) were obtained from PAN Biotech (Aidenbach, Germany).

### Cell culture

A549 human lung carcinoma cells were purchased from DSMZ (Deutsche Sammlung von Mikroorganismen und Zellkulturen GmbH, Braunschweig, Germany; A549: DSMZ no.: ACC 107, species confirmation as human with IEF of MDH, NP; fingerprint: multiplex PCR of minisatellite markers revealed a unique DNA profile). H358 cells were purchased from ATCC-LGC (Wesel, Germany; ATCC™ Number: CRL-5807™; cell line confirmation by cytogenetic analysis).

Cells were cultured in DMEM supplemented with 10% heat-inactivated FCS, 100 U/ml penicillin and 100 μg/ml streptomycin. Cells were grown in a humidified incubator at 37°C and 5% CO_2_. All incubations with test substances were performed in serum-free DMEM. Lovastatin lactone, NS-398 and GW9662 were dissolved in DMSO and diluted with PBS. Maximal DMSO content in experiments with substance combinations did not exceed 0.2% (v/v) DMSO. As vehicle control PBS containing the respective concentration of DMSO was used. Salts of lovastatin acid and mevalonic acid were dissolved in medium. Following resuscitation of frozen cultures none of the cell lines was cultured longer than 6 months.

### SiRNA transfections

Cells were seeded in 24-well plates at a density of 1 × 10^5^ cells per well (DNA fragmentation assays; Figure 8C, 8D), in 6-well plates at a density of 2 × 10^5^ cells per well (Western blot analyses, Figure 8A, 8B, lower panel) and in 96-well plates at a density of 5 × 10^3^ cells per well (WST-1 tests; Figure 8A, 8B, upper panel), and were allowed to adhere for 2-3 h. Transfection was performed as described previously [26, 28, 29]. In brief, cells were transfected with an equal ratio (w/v) of RNA to transfection reagent for 24 h in 10% DMEM prior to incubation with lovastatin lactone. Subsequently, cells were washed with PBS, transfected again in serum-free DMEM to provide constant transfection conditions, and incubation with vehicle or lovastatin lactone was started. Transfections were carried out using RNAiFect^®^ as transfection reagent (Qiagen, Hilden, Germany). SiRNA was obtained from Qiagen. The nonsilencing negative control RNA was from Eurogentec (Cologne, Germany). Final concentrations of COX-2 siRNA and non-silencing siRNA were 2.5 μg/ml, respectively.

### Quantitative reverse-transcriptase polymerase chain reaction

For quantitative real-time RT-PCR, cells were seeded in 24-well plates at a density of 1 × 10^5^ cells per well, grown for 24 h, and subsequently incubated with vehicle or test substances for the indicated time periods. COX-2 mRNA levels were determined by quantitative real-time RT-PCR using the TaqMan^®^ RNA-to-CT™1-Step Kit and TaqMan^®^ Gene Expression Assays for COX-2 mRNA analyses (Applied Biosystems, Darmstadt, Germany) as described previously [26, 29].

### Western blot analysis

For Western blot analyses, A549 or H358 cells were seeded in 6-well plates at a density of 2 × 10^5^ cells per well, grown for 24 h, and subsequently incubated with vehicle or test substances for the indicated time periods. Proteins were isolated and analysed as described previously [26, 28, 29]. In brief, following incubation, cells were washed with PBS, harvested and lysed in solubilization buffer (50 mM HEPES, 150 mM NaCl, 1 mM EDTA, 1% (v/v) Triton^®^ X-100, 10% (v/v) glycerol, 1 mM PMSF, 1 mM orthovanadate, 1 μg/ml leupeptin, 10 μg/ml aprotinin). Lysates were centrifuged at 10,000 × g for 5 min and supernatants were then used for Western blot analysis. Total protein of cell lysates was determined using the bicinchoninic acid assay (Pierce, Rockford, USA). Denatured proteins were separated using 10% sodium dodecyl sulfate polyacrylamide gels and then transferred to nitrocellulose membranes (Roth, Karlsruhe, Germany) that were blocked with 5% milk powder (BioRad, Munich, Germany). Membranes were probed with antibodies raised to COX-2 (BD Biosciences, Heidelberg, Germany), PPARγ (Santa Cruz, Heidelberg, Germany), Δ-actin (Sigma-Aldrich), lamin B1 (Abcam, Cambridge, UK), caspase-3, cleaved caspase-3, PARP, cleaved PARP (Cell Signaling Technology, Leiden, Netherlands) as well as horseradish peroxidase-conjugated Fab-specific anti-mouse (New England Biolabs GmbH, Frankfurt/Main, Germany) and anti-rabbit IgG (Cell Signaling Technology) as secondary antibody. Antibody binding was visualized by a chemiluminiferous solution (100 mM Tris-HCl pH 8.5, 1.25 mM luminol, 200 μM p-coumaric acid, 0.09% [v/v] H_2_O_2_). Densitometric analysis of band intensities was achieved by optical scanning and quantifying using the Quantity One 1-D Analysis Software (Biorad, Munich, Germany). For quantification of cell lysates all densitometric analyses were normalized to Δ-actin. Densitometric quantification of nuclear PPARγ was carried out by normalization to lamin B1. Apoptosis parameters (Figure 1C, right side) do not contain densitometric data due to the hardly detectable bands of cleaved caspase-3 in vehicle-treated A549 and H358 cells and of cleaved PARP in vehicle-treated A549 cells.

### Analyses of nuclear PPARγ by Western blot

For Western blot analyses of nuclear PPARγ cells were seeded in 10-cm dishes at a density of 2 × 10^6^ cells per well, grown for 24 h, and subsequently incubated with vehicle or test substances for the indicated time periods. Analysis of nuclear PPARγ was performed as previously as previously described [33] with slight modifications. In brief, following incubation, cells were suspended in PBS containing 6.2 mM NaF, 12.7 mM Δ-glycerophosphate, 15.5 mM p-nitrophenyl phosphate and 0.84 mM sodium orthovanadate. After a centrifugation step, pellets were resuspended in 1 ml of a hypotonic buffer (20 mM HEPES, pH 7.5, 5 mM NaF, 10 μM sodium molybdate, 0.1 mM EDTA). Afterwards, cells were allowed to swell on ice for 15 min, and 50 μl of a 10% (w/v) Nonidet^®^ P-40 solution was added to each tube. Following centrifugation of the homogenate, supernatants were carefully rinsed, and nuclear pellets were resuspended in 60 μl of complete lysis buffer containing 50 mM HEPES, pH 7.4, 150 mM NaCl, 1 mM EDTA, 1% (v/v) Triton^®^ X-100, 10% (v/v) glycerol, 0.5 mM DTT and 0.1% (w/v) SDS. Thereafter, tubes were shaken on ice for 30 min and a debris spin out was performed by centrifugation at 14,000 × g for 10 min. Supernatants were used for determination of nuclear protein by Western blot as described under Western blot analysis. As immunochemical characterisation of nuclear origin the membranes were rehybridized using a lamin B1 antibody.

### Analysis of cytosol-to-nucleus translocation of PPARγ by fluorescence microscopy

For visualization and quantification of nuclear PPARγ, cells were seeded in BD Falcon 4-well culture slides (BD Biosciences) at a density of 1 × 10^5^ cells per chamber and grown for 24 h. Following incubation with test substances or vehicle, cells were washed and fixed in 4% (v/v) formaldehyde. Subsequently, fixed cells were incubated with a PPARγ antibody (Biomol GmbH, Hamburg, Germany) and a lamin A/C antibody (New England Biolabs GmbH, Frankfurt/Main, Germany) for detection of cell nuclei. Secondary antibodies were a goat anti-rabbit Alexa Fluor^®^ 555 labelled IgG for detection of PPARγ and a goat anti-mouse Alexa Fluor^®^ 488 labelled IgG for detection of lamin A/C (Life Technologies Corporation, Darmstadt, Germany). All antibodies were diluted in PBS containing 0.3% (v/v) Triton^®^ X-100 and 1% (v/v) FCS. Cells were observed under a fluorescence microscope (Axio Scope. A1, Carl Zeiss Microscopy GmbH, Jena, Germany). Shapes of nuclear regions were merged to images of PPARγ-stained cells. Fluorescence intensity of PPARγ within lamin A/C-positive spots was quantified for 20 nuclei per sample. All images were analyzed using ZEN 2012 software from Zeiss (Jena, Germany).

### Determination of COX-2-dependently synthesized PGs

Cells seeded in 24-well plates at a density of 2 × 10^5^ cells per well and grown for 24 h were preincubated with NS-398 (1 μM) or its vehicle for 1 h. Thereafter, cells were incubated with vehicle or lovastatin lactone in the presence or absence of NS-398 for another 24 h. The final volume of the supernatant was 300 μl per well. Afterwards, cell culture media were removed and analyzed for PGE_2_, PGD_2_ and 15d-PGJ_2_ using enzyme immunoassay kits (PGE_2_, PGD_2_: Cayman Chemical, Ann Arbor, MI, USA; 15d-PGJ_2_: Enzo Life Sciences). For indication of percent control PG levels were normalized to whole cell protein and subsequently expressed as percent of vehicle control (100%).

### Cell viability and DNA fragmentation

Cells seeded at a density of 5 × 10^3^ cells per well in 96-well flat-bottom microplates (viability) or at 1 × 10^5^ cells per well in 24-well plates (DNA fragmentation) and grown for 24 h were used for incubations. Cell viability and DNA fragmentation were analysed using WST-1 test and Cell Death Detection ELISA^PLUS^ kit (both from Roche Diagnostics, Mannheim, Germany) according to the manufacturer's instructions, respectively.

### Determination of lovastatin forms in culture media and lysates of lovastatin-treated cells

For HPLC analyses 5 × 10^6^ cells (A549, H358) were seeded in 10-cm cell culture dishes and cultured for 24 h. For determination of extracellular lovastatin, 500 μl of acetonitrile were added to 500 μl of medium. The mixture was centrifuged for 7 min (20,000 × g) before chromatographic analysis. For determination of intracellular lovastatin, cells were trypsinized, centrifuged, and lysed by addition of 255 μl of water and sonification. An aliquot of 5 μl was used for protein determination. Finally, 250 μl acetonitrile were added for chromatographic determination using 25 μM mevastatin as internal standard. HPLC analyses were performed using a Prominence system (Shimadzu Deutschland GmbH, Duisburg, Germany) consisting of two high-pressure binary gradient pumps (LC-20AD) and a diode array detector (Nexera X2 SPD-M30A). The chromatographic separation was carried out at 30°C on a Multospher^®^120 column (RP 18, AQ-5μm, 250 × 3 mm, CS-Chromatographie Service GmbH, Langerwehe, Germany) with a precolumn (RP 18, AQ-5μm, 20 × 3 mm, CS-Chromatographie Service GmbH, Germany) by a gradient elution using (A) 0.1% (m/V) trifluoroacetic acid in bidistilled water and (B) acetonitrile: 34% A and 66% B, linear increase to 99% B in 15 min, holding for 4 min. The detection of the analytes was performed by UV absorbance at 240 nm. As the samples, calibration standards (0.1 up to 250 μmol/l) were likewise prepared with bidistilled water for measurements of intracellular lovastatin or with DMEM for extracellular lovastatin concentrations.

### Statistics

Comparisons between groups were performed with Student's two-tailed *t* test or with one-way ANOVA plus post hoc Bonferroni or Dunnett test using GraphPad Prism 5.00 (GraphPad Software, San Diego, CA). IC_50_ values were calculated by nonlinear regression of log(inhibitor) *vs*. response using least squares as fitting method in a 4 parameter calculation with variable slope. Concentrations (X) were transformed into log(X). Nonlinear regression was calculated by the formula: Y = Bottom + (Top-Bottom)/(1+10^((LogIC50-X)*HillSlope)). Bottom and top are plateaus of minimal or maximal loss of viability in response to the concentrations (X). Hill slope denotes steepness of the response of the cells toward rising concentrations of statins, i.e., loss of viability. IC_50_ represents loss of viability halfway between bottom and top.

## Abbreviations

COX-2: cyclooxygenase-2
HMG-CoA: 3-hydroxy-3-methylglutaryl coenzyme A
PG: prostaglandin
PPARγ: peroxisome proliferator-activated receptor γ
